# Influence of pancreatic fistula on survival after upfront pancreatoduodenectomy for pancreatic ductal adenocarcinoma: multicentre retrospective study

**DOI:** 10.1093/bjsopen/zrae125

**Published:** 2024-10-25

**Authors:** Fanny Castanet, Jeanne Dembinski, Bastien Cabarrou, Jonathan Garnier, Christophe Laurent, Nicolas Regenet, Antonio Sa Cunha, Charlotte Maulat, Laurence Chiche, Gabriella Pittau, Nicolas Carrère, Jean-Marc Regimbeau, Olivier Turrini, Alain Sauvanet, Fabrice Muscari

**Affiliations:** Hepato-Biliary-Pancreatic Surgery Unit, Digestive Surgery Department, University Hospital, Toulouse, France; Hepato-Biliary-Pancreatic Surgery Department, Beaujon Hospital, Clichy, France; Digestive Surgery Department, Amiens Picardie University Hospital, Amiens, France; Biostatistics and Health Data Science Unit, Institut Claudius Regaud, IUCT-O, Toulouse, France; Digestive Surgery Department, Paoli Calmette Institute, Marseille, France; Hepato-Biliary-Pancreatic Surgery Unit, Digestive Surgery Department, Bordeaux University Hospital, Bordeaux, France; Digestive Surgery Department, Nantes University Hospital, Nantes, France; Hepato-Biliary-Pancreatic Surgery Department, Paul Brousse Hospital, Clichy, France; Hepato-Biliary-Pancreatic Surgery Unit, Digestive Surgery Department, University Hospital, Toulouse, France; Hepato-Biliary-Pancreatic Surgery Unit, Digestive Surgery Department, Bordeaux University Hospital, Bordeaux, France; Hepato-Biliary-Pancreatic Surgery Department, Paul Brousse Hospital, Clichy, France; Hepato-Biliary-Pancreatic Surgery Unit, Digestive Surgery Department, University Hospital, Toulouse, France; Digestive Surgery Department, Amiens Picardie University Hospital, Amiens, France; Digestive Surgery Department, Paoli Calmette Institute, Marseille, France; Hepato-Biliary-Pancreatic Surgery Department, Beaujon Hospital, Clichy, France; Hepato-Biliary-Pancreatic Surgery Unit, Digestive Surgery Department, University Hospital, Toulouse, France

## Abstract

**Background:**

The effects of postoperative pancreatic fistula on survival rates remain controversial. The aim of the present study was to evaluate the influence of postoperative pancreatic fistula on overall survival and recurrence-free survival after upfront pancreatoduodenectomy for pancreatic ductal adenocarcinoma.

**Methods:**

Patients operated on between January 2007 and December 2017 at seven tertiary pancreatic centres for pancreatic ductal adenocarcinoma were included in the study. Postoperative pancreatic fistula was defined using the 2016 International Study Group on Pancreatic Surgery grading system. The impact of postoperative pancreatic fistula on overall survival, recurrence-free survival (excluding 90-day postoperative deaths) and corresponding risk factors were investigated by univariable and multivariable analyses. Comparisons between groups were made using the chi-squared or Fisher’s exact test for categorical variables and the Mann–Whitney U test for continuous variables. Odds ratios were estimated with their 95% confidence intervals. Survival rates were calculated using the Kaplan–Meier method with their 95% confidence intervals.

**Results:**

A total of 819 patients were included between 2007 and 2017. Postoperative pancreatic fistula occurred in 14.4% (*n* = 118) of patients; of those, 7.8% (*n* = 64) and 6.6% (*n* = 54) accounted for grade B and grade C postoperative pancreatic fistula respectively. The 5-year overall survival was 37.0% in the non-postoperative pancreatic fistula group and 45.3% in the postoperative pancreatic fistula cohort (*P* = 0.127). Grade C postoperative pancreatic fistula (excluding 90-day postoperative deaths) was not a prognostic factor for overall survival. The 5-year recurrence-free survival was 26.0% for patients without postoperative pancreatic fistula and 43.7% for patients with postoperative pancreatic fistula (*P* = 0.003). Eight independent prognostic factors for recurrence-free survival were identified: age over 70 years, diabetes, moderate or poor tumour differentiation, T3/T4 tumour stage, lymph node positive status, resection margins R1, vascular emboli and perineural invasion.

**Conclusion:**

This high-volume cohort showed that grade C postoperative pancreatic fistula, based on the 2016 International Study Group on Pancreatic Surgery grading system, does not impact overall or recurrence-free survival (excluding 90-day postoperative deaths).

## Teaser

The aim of this study was to evaluate the influence of postoperative pancreatic fistula on overall survival and recurrence-free survival after pancreatoduodenectomy for adenocarcinoma. This high-volume cohort showed that grade C postoperative pancreatic fistula, based on the 2016 International Study Group on Pancreatic Surgery classification system, does not impact overall or recurrence-free survival (excluding 90-day postoperative deaths).

## Introduction

The mortality rate for the pancreatoduodenectomy (PD) procedure is less than 5% in tertiary centres^1^. Furthermore, the morbidity rate remains high at 30–60%^1^, with the main complication being postoperative pancreatic fistula (POPF). The incidence of POPF varies from 3 to 45% with a mortality rate of 2.6% for all grades but reaching up to 25.7% for grade C, mainly due to bleeding complications^2–4^. There is no clear evidence of the effectiveness of any prevention methods^5^.

Long-term oncology outcomes after PD show an overall survival (OS) of approximately 20% at 5 years with a recurrence rate of 70–75% within 2 years despite 6 months of well conducted adjuvant therapy^6^. Recognized prognostic factors for survival are tumour related: diameter, differentiation, lymph node invasion and surgical margins^7^. Other factors, notably surgical complications including the occurrence of POPF have been mentioned^8,9^. Postoperative complications influence the patient’s eligibility to receive adjuvant chemotherapy^10^. Another hypothesis is that the local inflammation and systemic immunosuppression caused by these complications influence recurrence and therefore survival^11,12^. The link between postoperative fistula and medium-term recurrence has already been identified in other cancers such as rectal^13^, oesophageal^14^ or gastric^15^. With regard to pancreatic surgery, several authors have looked at the oncological effects of POPF but this correlation remains controversial^8,9,11,12,16,17^. The impact of POPF on OS and recurrence-free survival (RFS) remains unknown^8,16,17^. Some studies have shown that POPF is a significant predictor of peritoneal recurrence^12^, and that grade C POPF and postoperative complications have a negative impact on survival^9,10^.

Almost all studies carried out on the subject used the old classification system (International Study Group on Pancreatic Surgery (ISGPS) 2005). Under the new definition (ISGPS 2016), the incidence of clinically relevant postoperative pancreatic fistula (grade B or C fistula) after PD varies between 1 and 36%^18,19^. The aim of this work was to evaluate the impact of grades B and C POPF according to the 2016 ISGPS grading system on OS and RFS after upfront PD for pancreatic ductal adenocarcinoma (PDAC).

## Methods

### Patients

This was a French retrospective multicentre study which involved seven tertiary pancreatic centres between January 2007 and December 2017. Every patient who underwent PD for a pathologic confirmed PDAC was included (TNM 8th edition UICC)^20^.

Exclusion criteria were R2 resection, PD with arterial reconstructions, intraoperative metastatic disease, neoadjuvant chemotherapy, neoadjuvant radiotherapy, postoperative radiotherapy and PD without restoration of pancreatic continuity.

The data were collected retrospectively from electronic and paper records and included patient demographics, body mass index, co-morbidities, type of surgery performed, intraoperative pancreatic texture, diameter of the main pancreatic duct, estimated intraoperative blood loss, type of drainage performed, whether a transanastomotic stent was placed, duration of hospital stay, histological findings, resection margin, perineural and perivascular invasion, complications graded according to Clavien–Dindo classification^21^, adjuvant treatment and tumour recurrence.

### Ethics

The study was conducted in accordance with the Declaration of Helsinki (as revised in 2013). The study was approved by the Toulouse University Hospital Institutional Ethics Board (No: RnIPH 2021–57).

### Follow-up

All patients attended a multidisciplinary oncology consultation meeting and were followed up with computer tomography (CT) every 3 months for 3 years, then every 6 months to 1 year for 2 years, accounting for an overall follow-up interval of 5 years. The follow-up was updated until 31 December 2019 through medical records and/or telephone interviews. Patients were followed up from the date of surgery until their final oncology check-up or death.

### Definitions

POPF and its severity stages were defined by the 2016 ISGPS grading system. Recurrence was classified as local (resection site), lymph node, metastatic or multisite and confirmed by imaging. OS was defined as the time between the date of surgery and the date of death from any cause. Living patients were censored at the time of last follow-up. RFS was interpreted as the time between the date of surgery and the date of recurrence or death from any cause. RFS and OS analysis was performed by excluding 90-day postoperative deaths (Clavien–Dindo V).

### Statistical analysis

Data were summarized by frequency and percentage for categorical variables and by median and range for continuous variables. Comparisons between groups were made using the chi-squared or Fisher’s exact test for categorical variables and the Mann–Whitney U test for continuous variables. Odds ratios (OR) were estimated with their 95% confidence intervals (c.i.). Survival rates were calculated using the Kaplan–Meier method with their 95% c.i. Univariable and multivariable analyses were performed using the Logrank test and Cox proportional hazards model. Hazard ratios (HR) were calculated with their 95% c.i. Statistically significant variables in univariable analysis and/or deemed clinically relevant were included in the multivariable analyses. All statistical tests were two-sided and a *P* value less than 0.05 was considered statistically significant. Statistical analyses were conducted using STATA v. 16 (StataCorp, College Station, TX, USA) software.

## Results

A total of 819 patients were included. All PD were performed by open approach. A total of 118 patients (14.4%) presented clinically relevant POPF: 7.8% with grade B fistula (*n* = 64) and 6.6% with grade C fistula (*n* = 54).

### Pre- and intraoperative criteria

The median age of this cohort was 66 years (range 36–88). Biliary drainage was performed in 56.7% of patients (*n* = 455). Preoperative criteria strongly associated with POPF in univariable analysis included: male sex, slight weight loss, no diabetes, ASA score, biliary drainage and no jaundice at the time of diagnosis (*P* < 0.05) (*Table 1*).

**Table 1:** Preoperative and intraoperative criteria (univariable analysis)

	Total (*n* = 819)	No POPF (*n* = 701)	POPF (*n* = 118)	*P*
**Sex**				0.001
Male	453 (55.3)	364 (51.9)	89 (75.4)	
Female	366 (44.7)	337 (48.1)	29 (24.6)	
Age over 70 years	287 (35.0)	251 (35.8)	36 (30.5)	0.264
Weight loss ≥10% (MD)	207 (27.5) (67)	187 (29.0) (57)	20 (18.5) (10)	**0.023**
Smoking (MD)	181 (22.3) (6)	150 (21.6) (6)	31 (26.3)	0.258
Diabetes (MD)	189 (23.7) (20)	173 (25.3) (17)	16 (13.9) (3)	**0.008**
ASA score II–III (MD)	630 (77.8) (9)	553 (79.9) (9)	77 (65.3)	**0.001**
Preoperative jaundice	507 (61.9)	445 (63.5)	62 (52.5)	**0.024**
**Biliary drainage (MD)**	455 (56.7) (16)	401 (58.3) (13)	54 (47.0) (3)	**0.023**
Endoscopic (MD)	426 (54.1) (31)	376 (55.5) (24)	50 (45.0) (7)	**0.040**
Percutaneous (MD)	16 (2.0) (29)	15 (2.2) (23)	1 (0.9) (6)	0.714
**Pancreatic anastomosis (MD)**	(2)	(2)	–	0.780
Pancreatojejunostomy	664 (81.3)	567 (81.1)	97 (82.2)	
Pancreatogastrostomy	153 (18.7)	132 (18.9)	21 (17.8)	
Soft pancreas (MD)	263 (33.0) (22)	199 (29.1) (18)	64 (56.1) (4)	**<0.001**
Non-dilated Wirsung (<3 mm) (MD)	300 (39.0) (49)	236 (35.6) (39)	64 (59.3) (10)	**<0.001**
Blood loss (ml), median (range) (MD)	400 (0–7820) (117)	350 (0–7820) (99)	400 (50–4000) (18)	0.066
Blood transfusion (MD)	140 (18.0) (108)	112 (16.8) (96)	28 (24.6) (12)	**0.047**
Venous resection (MD)	212 (29.7) (105)	196 (32.3) (94)	16 (15.0) (11)	**0.001**
**Abdominal drainage (MD)**	(5)	(4)	(1)	0.482
None	20 (2.5)	17 (2.4)	3 (2.6)	
Suction	106 (13.0)	87 (12.5)	19 (16.2)	
Non-suction	688 (84.5)	593 (85.1)	95 (81.2)	

Values are *n* (%) unless otherwise stated. *P* < 0.05 are in bold. ASA, American Society of Anesthesiologists; MD, missing data; POPF, postoperative pancreatic fistula.

In 81.3% (*n* = 664) of the patients, the pancreatic anastomosis involved a pancreatic jejuno (PJ) anastomosis. The pancreas was considered soft (normal) in 33.0% of patients (*n* = 263) while the Wirsung duct was not dilated in 39.0% of patients (*n* = 300). A venous resection was associated with the procedure in 29.7% of patients (*n* = 212).

### Postoperative and anatomopathological criteria

The 90-day postoperative mortality rate in this cohort was 3.5% (*n* = 29) with 19.5% of patients who experienced POPF (*n* = 23) *versus* 0.9% (*n* = 6) who did not. The 90-day surgical complication rate was 50.8% (*n* = 416). The rate of delayed gastric emptying amounted to 24.4% (*n* = 200) but was significantly higher in the presence of POPF: 39.0% (*n* = 46) *versus* 22.0% (*n* = 154) (*P* < 0.001). The haemorrhage rate was 11.6% (*n* = 95) but increased considerably with POPF: 50.8% (*n* = 60) *versus* 5.0% (*n* = 35) (*P* < 0.001).

The median duration of hospital stay was 17 days (range 2–379) but considerably longer if POPF was present: 29 days *versus* 15 days (*P* < 0.001).

The specimen analysis revealed that patients in the POPF group experienced fewer vascular emboli (44.1% (*n* = 52) *versus* 54.7% (*n* = 377), *P* = 0.0322) and more well differentiated tumours (50.0% (*n* = 55) *versus* 37.3% (*n* = 248), *P* = 0.0114). The lymph node positive status rate was 57.6% (*n* = 68) in the POPF group *versus* 65.9% (*n* = 462) in the non-POPF cohort (*P* = 0.0817) (*Table 2*).

**Table 2:** Histological and postoperative data

	Total (*n* = 819)	No POPF (*n* = 701)	POPF (*n* = 118)	*P*
**Tumour differentiation (MD)**	(44)	(36)	(8)	**0.011**
Well	303 (39.1)	248 (37.3)	55 (50.0)	
Moderate/low	472 (60.9)	417 (62.7)	55 (50.0)	
**Tumour stage (MD)**	(3)	(3)	–	0.406
T1–2	190 (23.3)	159 (22.8)	31 (26.3)	
T3–4	626 (76.7)	539 (77.2)	87 (73.7)	
Positive lymph node status	530 (64.7)	462 (65.9)	68 (57.6)	0.082
R1 Resection margin (MD)	237 (29.0) (3)	206 (29.5) (2)	31 (26.5) (1)	0.512
Vascular emboli (MD)	429 (53.2) (12)	377 (54.7) (12)	52 (44.1)	**0.032**
Perineural invasion (MD)	611 (75.6) (11)	529 (76.7) (11)	82 (69.5)	0.093
**Clavien–Dindo grade**				**< 0.001**
0–II	630 (76.9)	618 (88.2)	12 (10.2)	
III–V	189 (23.1)	83 (11.8)	106 (89.8)	
Bleeding complications	95 (11.6)	35 (5.0)	60 (50.8)	**< 0.001**
Delayed gastric emptying	200 (24.4)	154 (22.0)	46 (39.0)	**< 0.001**
Surgical complications	416 (50.8)	302 (43.1)	114 (96.6)	**< 0.001**
Duration of hospital stay (days), median (range)	17 (2–379)	15 (2–379)	29 (8–184)	**< 0.001**
Adjuvant chemotherapy (MD)	614 (76.2) (13)	554 (80.1) (9)	60 (52.6) (4)	**< 0.001**
Recurrence	505 (61.7)	463 (66.0)	42 (35.6)	NA
**Localization of recurrence (MD)**	(3)	(3)	–	NA
Local	104 (20.7)	96 (20.9)	8 (19.0)	
Lymph node	30 (6.0)	28 (6.1)	2 (4.8)	
Metastatic	242 (48.2)	224 (48.7)	18 (42.9)	
Multisite	126 (25.1)	112 (24.3)	14 (33.3)	
**Treatment of recurrence (MD)**	(40)	(37)	(3)	NA
Chemotherapy	362 (77.8)	334 (78.4)	28 (71.8)	
Radiochemotherapy	32 (6.9)	29 (6.8)	3 (7.7)	
Surgical/thermoablation	35 (7.5)	34 (8.0)	1 (2.6)	
Supportive care	36 (7.7)	29 (6.8)	7 (17.9)	

Values are *n* (%) unless otherwise stated. *P* < 0.05 are in bold. POPF, postoperative pancreatic fistula; MD, missing data; NA, not applicable.

### Follow-up and recurrence

The median follow-up interval in this population was 60.6 months (95% c.i. 57.3 to 63.6). At the end of the study, 38.2% (*n* = 313) of patients were alive: 39.8% (*n* = 47) in the POPF group *versus* 37.9% (*n* = 266) in the non-POPF cohort. A total of 79.0% (*n* = 614) of patients received adjuvant chemotherapy: 65.9% (*n* = 60) in the POPF group *versus* 80.8% (*n* = 554) in the non-fistula group (*P* = 0.001) (*Table 2*).

The recurrence rate in the overall population was 61.7% (*n* = 505) (*Table 2*). Local and metastatic recurrence accounted for 20.7% (*n* = 104) and 48.2% (*n* = 242) of patients respectively. The rate of recurrence was 35.6% (*n* = 42) and 66.0% (*n* = 463) respectively in POPF patients and non-fistula patients.

### Overall survival

The OS analysis was performed by excluding 90-day postoperative deaths. The OS at 1, 3 and 5 years was 84.6% (95% c.i. 81.8 to 86.9), 53.0% (95% c.i. 49.3 to 56.5) and 37.9% (95% c.i. 34.2 to 41.7) respectively with a median of 39.9 months (95% c.i. 34.7 to 42.7).

**Fig. 1:**
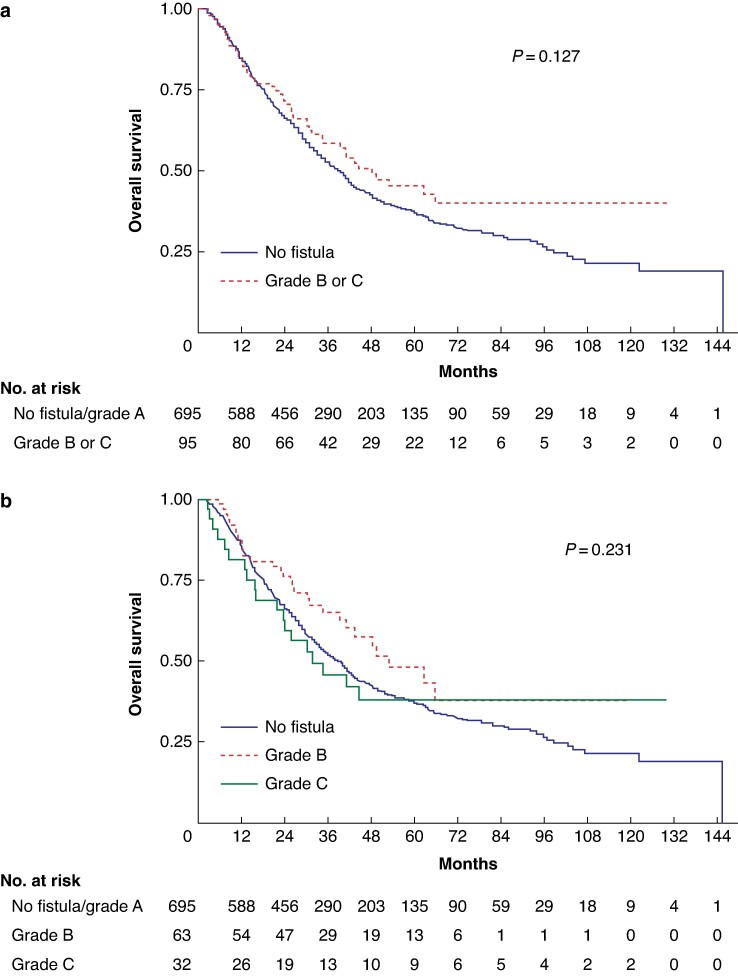
Overall survival (overall population without Clavien–Dindo grade V patients) **a** Population with or without postoperative pancreatic fistula (POPF). **b** Population without POPF, grade B POPF and grade C POPF

The 5-year OS was 37.0% (95% c.i. 33.0 to 41.0) in the non-POPF group (median of 38.9 months, 95% c.i. 33.9 to 42.1) and 45.3% (95% c.i. 33.7 to 56.1) in the group with POPF (median of 48.2 months, 95% c.i. 34.4 to not reached (NR)), *P* = 0.127 (*Fig. 1a*).

The 5-year OS was 48.1% (95% c.i. 32.6 to 62.0) in the group with grade B POPF (median of 52.7 months, 95% c.i. 39.1 to NR), and 38.1% (95% c.i. 21.1 to 55.0) in the group with grade C POPF (median of 31.5 months, 95% c.i. 15.8 to NR), *P* = 0.231 (*Fig. 1b*).

The univariable analysis is presented in *Table S1*. Six independent prognostic factors for OS were isolated in the multivariable analysis: age over 70 years, diabetes, T3/T4 tumour stage, lymph node positive status, adjuvant chemotherapy and perineural invasion (*Table 3*).

**Table 3:** Independent prognostic factors for overall survival without Clavien–Dindo grade V patients (multivariable analysis)

	HR	95% c.i.	*P*
**POPF^18^**			
Grade B	0.77	(0.49,1.20)	0.251
Grade C	0.95	(0.54,1.66)	0.851
Age ≥ 70 years	1.26	(1.03,1.53)	**0.023**
Diabetes	1.33	(1.07,1.65)	**0.010**
Moderate to poor differentiation	1.21	(0.99,1.48)	0.057
Tumour grade T3–T4^20^	1.43	(1.08,1.91)	**0.013**
Lymph node status N+ ^20^	1.96	(1.53,2.51)	**<0.001**
R1 resection	1.15	(0.94,1.41)	0.171
Vascular emboli	1.22	(0.99,1.49)	0.062
Perineural invasion	1.54	(1.17,2.04)	**0.002**
Adjuvant chemotherapy	0.60	(0.47,0.76)	**<0.001**

*P* < 0.05 are in bold. HR, hazard ratio; POPF, postoperative pancreatic fistula.

### Recurrence-free survival

RFS analysis was performed by excluding 90-day postoperative deaths.

The RFS of this population at 1, 3 and 5 years was 65.3% (95% c.i. 61.9 to 68.5), 35.0% (95% c.i. 31.6 to 38.4) and 28.0% (95% c.i. 24.6 to 31.4) with a median of 20.1 months (95% c.i. 18.0 to 22.6).

**Fig. 2:**
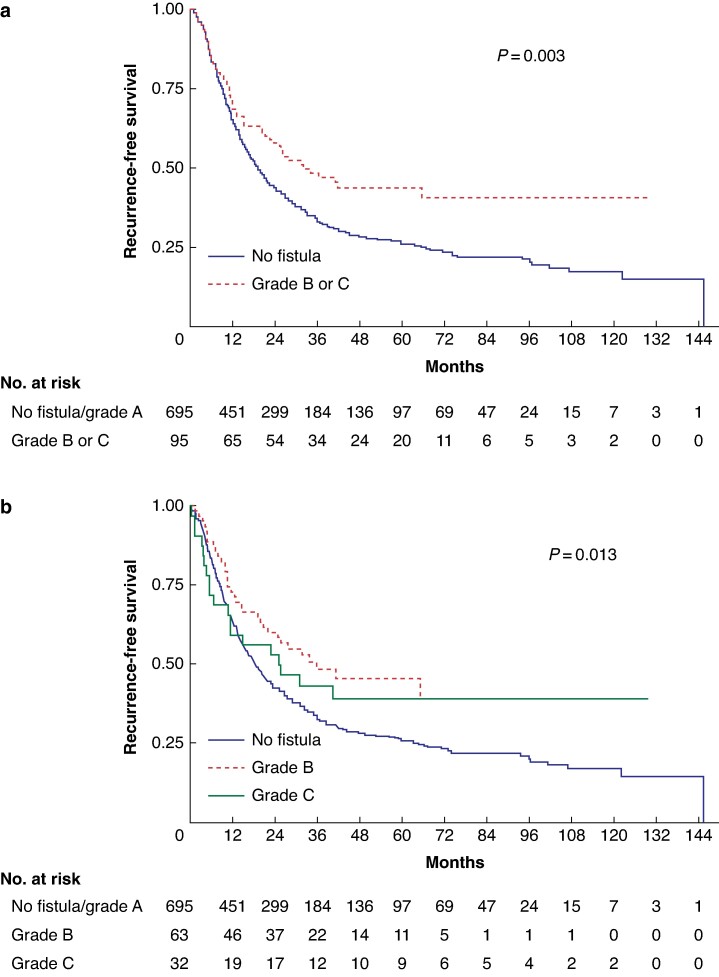
Recurrence-free survival (overall population without Clavien–Dindo grade V patients) **a** Population with or without postoperative pancreatic fistula (POPF). **b** Population without POPF, grade B POPF and grade C POPF

The 5-year RFS was 26.0% (95% c.i. 22.5 to 29.6) for patients without POPF (median of 19.1 months, 95% c.i. 16.9 to 21.5) and 43.7% (95% c.i. 32.9 to 53.9) for patients with POPF (median of 32.1 months, 95% c.i. 21.2 to NR), *P* = 0.003 (*Fig. 2a*). The 5-year RFS was 45.8% (95% c.i. 32.1 to 58.4) in the grade B POPF group (median of 36.3 months, 95% c.i. 21.2 to NR), and 39.3% (95% c.i. 22.3 to 55.9) in the group with grade C POPF (median of 25.6 months, 95% c.i. 7.2 to NR), *P* = 0.013 (*Fig. 2b*).

The univariable analysis is presented in *Table S2*. In the multivariable analysis, eight independent prognostic factors for RFS were isolated: age over 70 years, diabetes and tumour-related criteria which included differentiation, T3/4, lymph node positive status, resection margins R1, vascular emboli and perineural invasion (*Table 4*).

**Table 4:** Prognostic factors for recurrence-free survival without Clavien–Dindo grade V patients (multivariable analysis)

	HR	95% c.i.	*P*
**B or C pancreatic fistula^18^**	0.73	(0.51,1.05)	0.089
Type of fistula			
B fistula	0.65	(0.43,0.98)	0.041
C fistula	0.99	(0.57,1.71)	0.958
Clavien–Dindo III–IV^21^	1.14	(0.86,1.51)	0.347
Age over 70 years	1.21	(1.01,1.45)	**0.039**
Diabetes	1.26	(1.03,1.55)	**0.024**
Moderate to poor differentiation	1.28	(1.07,1.54)	**0.007**
Tumour grade T3–T4^20^	1.34	(1.04,1.72)	**0.022**
Lymph node status N+^20^	1.92	(1.55,2.39)	**<0.001**
R1 resection margin	1.23	(1.02,1.48)	**0.033**
Perineural invasion	1.45	(1.13,1.87)	**0.003**
Vascular emboli	1.25	(1.04,1.51)	**0.020**

*P* < 0.05 are in bold. HR, hazard ratio.

## Discussion

The results of this study are consistent with the literature^9,11,12,16,17,22^ which, for the most part, used the 2005 ISPGF classification system. The 2016 grading classification increased the incidence of grade B fistula and reduced the grade C rate which recentred on life-threatening POPFs^23^. The mortality rate found in the present analysis was identically reflected in the interventional arm of the Dutch stepped-wedge randomized PORSCH (The Care After Pancreatic Resection According to an Algorithm for Early Detection and Minimally Invasive Management of Pancreatic Fistula versus Current) trial^24^.

In this study, POPF had no influence on the recurrence rate or its location. The recurrence rate was lower than that identified in the literature, which is close to 80%^5^. This can be explained by the fact that this study was conducted in tertiary centres with a more rigorous selection of patients. The theory that POPFs influence recurrence is based on a proinflammatory phenomenon caused by leakage of pancreatic juice that could favour the implantation of tumour cells and thus lead to loco-regional recurrence^11,12,25^, as demonstrated in other cancers such as breast^26^ or rectal^13^.

The OS in this cohort was 37.9% at 5 years with a median of 39.9 months. These figures mirror the same values in the literature where the rate varies from 20 to 30%^27^. In this study, grade C POPF was not found to be a prognostic factor of OS. In the literature, some studies have reported that grade C POPF negatively impacted OS, but the authors used different classification systems for pancreatic fistula and they included 90-day postoperative deaths (Clavien–Dindo V)^8,9,16,17^.

The RFS in this cohort was 27.0% at 5 years with a median of 19 months. The median RFS in the literature is 12 to 19 months^9,28^. Grade C POPF was not a prognostic factor but influenced survival since the RFS curves did not differ significantly while the population of patients exhibited considerably fewer adverse histological criteria (differentiation, vascular emboli, positive node). Patients should perhaps be matched on the basis of these three histological aggressiveness criteria in order to study the real effect of POPF on RFS. Some studies in the literature^13,16,17^ found that POPF had an impact in terms of local recurrence but no effect on OS or RFS. One study^11^ demonstrated a negative effect on RFS caused by grade C POPF with the same explanations as for OS, namely mortality rate and less access to chemotherapy.

A recent study from The Netherlands showed that major complications (Clavien–Dindo grade III or higher) or organ failure were significantly associated with both reduced RFS and OS^10^. In this study, the negative effect of major complications on survival appeared to be partly mediated by the omission of adjuvant therapy. The European Study Group for Pancreatic Cancer (ESPAC-3 trial) demonstrated that completion of all cycles of planned adjuvant therapy rather than early initiation was an independent prognostic factor after pancreatic cancer resection^29^.

The main limit of this study was its retrospective nature. Indeed, some data could not be retrieved (for example the number of chemotherapy cycles). Nevertheless, the high patient volume allowed good quality statistical power to be maintained. This research constituted one of the largest series carried out on this subject. The population of this cohort was quite homogeneous because patients who received neoadjuvant treatment were excluded. This also introduced the bias that this analysis might have been different if neoadjuvant patients had also been included, which is now widely used.

This was one of the first studies on POPFs using the new 2016 POPF classification system which has enabled good homogeneity between centres compared with the former classification structure. In conclusion, this high-volume cohort showed that grade C POPF, based on the 2016 ISGPS classification system, does not impact OS or RFS (excluding 90-day postoperative deaths).

## Supplementary Material

zrae125_Supplementary_Data

## Data Availability

No additional data are available.
