# Supplementary material for: Influence of pancreatic fistula on survival after upfront pancreatoduodenectomy for pancreatic ductal adenocarcinoma: multicentre retrospective study
Source: BJS Open. 2024 Oct 25;8(5):zrae125. doi: 10.1093/bjsopen/zrae125 (PMC11505446; doi:10.1093/bjsopen/zrae125)
Supplement: zrae125_Supplementary_Data [file zrae125_supplementary_data.zip › Supplementary-Table-2R3.docx]

**Supplementary Table 2.** Prognostic factors for recurrence-free survival (excluding 90 days post-operative deaths: Clavien-Dindo V complication). Univariate analysis. BMI: Body mass index, ASA: American Society of Anesthesiologists.

| Median [95% CI] HR [95% CI] |
| --- |
| B or C pancreatic fistula (18): p = 0.0035  No 19.1 [16.9;21.5] 1.00  Yes 32.1 [21.2; .] 0.66 [0.49;0.87]    Type of fistula: p = 0.0126  No fistula 19.1 [16.9;21.5] 1.00  B Fistula 36.3 [21.2; .] 0.62 [0.43;0.88]  C Fistula 25.6 [ 7.2; .] 0.73 [0.46;1.16]    SEX: p = 0.3981  Male 21.0 [18.5;25.1 1.00  Female 18.0 [14.7;22.8] 1.07 [0.91;1.27]    Age at the time of surgery (years): p = 0.0063  < 70 years 22.5 [19.7;26.4] 1.00  >= 70 years 15.8 [13.7;19.7] 1.27 [1.07;1.50]    BMI (cl): p = 0.7531  <25 20.9 [17.9;26.0] 1.00  >=25 19.0 [15.7;24.1] 1.03 [0.86;1.23]    Weight loss(%): p = 0.5031  <10% 20.4 [17.1;25.1] 1.00  >=10% 20.8 [16.2;23.9] 1.07 [0.88;1.29]    Smoking: p = 0.8271  No 19.7 [17.0;22.0] 1.00  Yes 21.8 [16.7;27.8] 0.98 [0.80;1.19]    Diabetes: p = 0.0075  No 21.4 [18.1;25.3] 1.00  Yes 16.4 [12.9;21.0] 1.30 [1.07;1.57]    ASA: p = 0.0679  1 26.3 [19.7;32.5] 1.00  2-3 19.0 [16.4;21.6] 1.21 [0.99;1.48] |
| Surgical complications (abdominal infectious complications, hemorrhage, Delayed gastric empty, vascular thrombosis): p = 0.5727  No 19.4 [16.1;23.1] 1.00  Yes 20.5 [17.7;25.3] 0.95 [0.81;1.13]    Bleeding complications: p = 0.1050  No 19.7 [17.1;21.7] 1.00  Yes 29.3 [17.1; .] 0.66 [0.47;0.93]    Delayed Gastric empty: p = 0.2566  No 20.8 [18.0;23.7] 1.00  Yes 18.1 [14.8;24.0] 1.12 [0.92;1.35]    Medical complications (cardiac, pulmonary, infectious, metabolic, thromboembolic, and urinary complications): p = 0.1763  No 21.5 [19.0;25.1] 1.00  Yes 15.2 [13.7;21.0] 1.13 [0.94;1.36]    Clavien-Dindo (21): p = 0.0324  0-II 19.2 [16.9;22.0] 1.00  III-V 23.4 [18.1;36.3] 0.79 [0.63;0.98]    Tumour differentiation: p = 0.0002  Good 25.4 [21.5;31.2] 1.00  Avg/Low 15.7 [13.8;18.5] 1.40 [1.17;1.66]    T (20): p < 0.0001  T1-2 63.2 [39.1;71.6] 1.00  T3-4 16.4 [15.1;19.0] 2.13 [1.70;2.67]    N (20): p < 0.0001  N0 58.8 [36.3;71.6] 1.00  N+ 15.1 [13.6;16.9] 2.49 [2.05;3.02]    R status: p < 0.0001  R0 23.7 [19.7;28.3] 1.00  R1 14.4 [11.5;17.7] 1.54 [1.29;1.84]    Vascular emboli: p < 0.0001  No 31.5 [25.4;37.3] 1.00  Yes 15.2 [12.7;17.8] 1.75 [1.48;2.08]    Perineural invasion: p < 0.0001  No 63.5 [40.7; .] 1.00  Yes 16.3 [14.7;18.6] 2.30 [1.84;2.88] |
