# Supplementary material for: Influence of pancreatic fistula on survival after upfront pancreatoduodenectomy for pancreatic ductal adenocarcinoma: multicentre retrospective study
Source: BJS Open. 2024 Oct 25;8(5):zrae125. doi: 10.1093/bjsopen/zrae125 (PMC11505446; doi:10.1093/bjsopen/zrae125)
Supplement: zrae125_Supplementary_Data [file zrae125_supplementary_data.zip › Supplementary_Material.docx]

**Title:** Influence of Pancreatic Fistula on Survival after upfront Pancreaticoduodenectomy for Pancreatic ductal Adenocarcinoma: French Multicentre Retrospective Study.

**Authors:** Fanny Castanet^(1)^, Jeanne Dembiski^(2,3)^, Bastien Cabarrou^(4)^, Jonathan Garnier^(5)^, Christophe Laurent^(6)^, Nicolas Regenet^(7)^, Antonio Sa Cunha^(8)^, Charlotte Maulat^(1)^, Laurence Chiche^(6)^, Gabriella Pittau^(8)^, Nicolas Carrère^(1)^, Jean-Marc Regimbeau^(3)^, Olivier Turrini^(5)^, Alain Sauvanet^(2)^ and Fabrice Muscari^(1)^.

**Institutions:**

1- Hepato-Biliary-Pancreatic Surgery Unit, Digestive Surgery Department, Toulouse, France.

2- Hepato-Biliary-Pancreatic Surgery Department, Beaujon Hospital, Clichy, France.

3- Digestive Surgery Department, Amiens, France.

4- Biostatistics and Health Data Science Unit, Institut Claudius Regaud, IUCT-O, Toulouse, France.

5- Digestive Surgery Department, Paoli Calmette Institute, Marseille, France.

6- Hepato-Biliary-Pancreatic Surgery Unit, Digestive Surgery Department, Bordeaux, France.

7- Digestive Surgery Department, Nantes, France.

8- Hepato-Biliary-Pancreatic Surgery Department, Paul Brousse Hospital, Clichy, France.

**Corresponding Author:** Charlotte MAULAT, Service de Chirurgie Digestive et Transplantation, CHU Rangueil, 1 avenue Jean Poulhès, 31059 Toulouse, France.

Telephone: +33 561322088; Fax: +33 561322936; Email: maulat.c@chu-toulouse.fr

| **Supplementary Tables** |  |
| --- | --- |
| *Supplementary Table 1* | *page. 12* |
| *Supplementary Table 2* | *page. 13* |
|  |  |

**Supplementary Materials - Index**
