# Supplementary material for: Influence of pancreatic fistula on survival after upfront pancreatoduodenectomy for pancreatic ductal adenocarcinoma: multicentre retrospective study
Source: BJS Open. 2024 Oct 25;8(5):zrae125. doi: 10.1093/bjsopen/zrae125 (PMC11505446; doi:10.1093/bjsopen/zrae125)
Supplement: zrae125_Supplementary_Data [file zrae125_supplementary_data.zip › Supplementary-Table-1R3.docx]

**Supplementary Table 1**. Prognostic factors for overall survival (excluding 90 days post-operative deaths: Clavien-Dindo V complication). Univariate analysis. BMI: Body mass index, ASA: American Society of Anesthesiologist.

Median [95%CI] HR [95%CI] P-value

B or C pancreatic fistula (18): p = 0.1267

No 38.9 [33.9;42.1] 1.00

Yes 48.2 [34.4; .] 0.79 [0.59;1.07]

Type of fistula : p = 0.2306

No fistula 38.9 [33.9;42.1] 1.00

B Fistula 52.7 [39.1; .] 0.72 [0.50;1.05]

C Fistula 31.5 [15.8; .] 0.93 [0.59;1.48]

Sex : p = 0.8803

Male 40.6 [33.9;45.9] 1.00

Female 37.7 [32.8;44.2] 1.01 [0.85;1.22]

Age at the time of surgery (years): p = 0.0006

< 70 years 42.1 [37.7;48.7] 1.00

>= 70 years 32.0 [25.3;40.1] 1.38 [1.15;1.65]

BMI (cl) : p = 0.8661

<25 41.7 [36.7;48.2] 1.00

>=25 40.4 [32.8;46.0] 1.02 [0.84;1.23]

Weight loss (%): p = 0.3954

<10% 41.0 [36.4;47.4] 1.00

>=10% 40.3 [33.1;48.3] 1.09 [0.89;1.35]

Smoking: p = 0.8943

No 39.8 [34.4;43.8] 1.00

Yes 40.3 [29.8;47.8] 1.01 [0.82;1.26]

Diabetes: p = 0.0046

No 41.0 [36.8;47.8] 1.00

Yes 33.1 [26.3;40.9] 1.35 [1.10;1.66]

ASA classification: p = 0.1111

1 43.8 [34.4;60.4] 1.00

2-3 39.1 [33.7;42.3] 1.20 [0.96;1.49]

Surgical complications (abdominal infectious complications, hemorrhage, Delayed gastric empty, vascular thrombosis): p = 0.8670

No 40.3 [34.6;46.0] 1.00

Yes 39.2 [33.1;44.3] 1.02 [0.85;1.22]

Bleeding complications: p = 0.1195

No 39.2 [34.4;42.1] 1.00

Yes 48.2 [30.1; .] 0.76 [0.53;1.08]

Delayed gastric empty: p = 0.0868

No 40.4 [35.6;45.9] 1.00

Yes 36.2 [29.7;42.5] 1.20 [0.97;1.47]

Medical complications (cardiac, pulmonary, infectious, metabolic, thromboembolic, and urinary complications): p = 0.1740

No 40.9 [35.8;46.3] 1.00

Yes 35.2 [28.0;41.7] 1.15 [0.94;1.40]

Clavien-Dindo (21): p = 0.9230

0-II 39.8 [34.7;43.0] 1.00

III-IV 40.1 [30.1;49.3] 0.99 [0.79;1.24]

Tumour differentiation: p = 0.0004

Well 47.8 [39.2;62.4] 1.00

Moderate/Poor 33.4 [28.8;37.9] 1.42 [1.17;1.72]

T (20): p < 0.0001

T1-2 81.7 [63.5;102.2] 1.00

T3-4 33.3 [30.0;36.7] 2.16 [1.67;2.79]

N (20): p < 0.0001

N0 74.6 [63.2; .] 1.00

N+ 30.0 [27.7;33.4] 2.33 [1.89;2.88]

R status: p < 0.0001

R0 42.7 [38.9;49.9] 1.00

R1 29.6 [23.4;36.4] 1.49 [1.23;1.80]

Vascular emboli: p < 0.0001

No 50.0 [42.1;65.3] 1.00

Yes 30.1 [27.6;34.6] 1.66 [1.38;2.00]

Perineural invasion: p < 0.0001

No 81.7 [63.5; .] 1.00

Yes 33.4 [30.0;37.0] 2.18 [1.70;2.78]

Adjuvant chemotherapy : p = 0.0318

No 31.5 [20.9;40.4] 1.00

Yes 40.7 [35.6;44.7] 0.79 [0.63;0.98]
